# Stress Reactivity in Chronic Tinnitus

**DOI:** 10.1038/srep41521

**Published:** 2017-01-30

**Authors:** Linda T. Betz, Andreas Mühlberger, Berthold Langguth, Martin Schecklmann

**Affiliations:** 1 University of Regensburg, Department of Psychiatry and Psychotherapy, Regensburg, Germany; 2 University of Regensburg, Department of Psychology (Clinical Psychology and Psychotherapy), Regensburg, Germany

## Abstract

Tinnitus is primarily an auditory symptom. Yet not only patients and clinicians, but also current pathophysiological models relate the onset and maintenance of tinnitus to stress. Here physiological and psychological stress reactivity was investigated in 19 patients with subjective chronic tinnitus and 19 comparable healthy controls. All participants underwent five consecutive measurements in one session including three resting conditions and two stress tasks in between (mental arithmetic and concentration on tinnitus/ear noise). Stress reactivity was assessed by heart rate (HR), heart rate variability (HRV) and subjective ratings for each of the five measurements. In patients with tinnitus, mean HR was overall decreased and blunted in response to acute stress induced by mental arithmetic compared to controls. HRV measures did not differ between both groups. Tinnitus sufferers indicated more subjective stress and increased awareness of tinnitus after the mental arithmetic task (during both resting and concentration on tinnitus measurements), but perceived similar levels of stress during mental arithmetic stress. In contrast to controls, HR and HRV were not correlated and also strain reports and physiological data were not associated in tinnitus. Our data show hints for a de-synchronization of physiological and psychological stress reactivity in chronic tinnitus.

About one fourth of tinnitus sufferers in Germany considers stress as the main reason for their tinnitus^1^,^2^. Similarly, many clinicians relate tinnitus onset and maintenance not only to cochlear mechanisms, but also to stress^3^. Despite the suggestion of a link between stress and tinnitus, there is little empirical support for this idea to date. Several issues are discussed in the association of tinnitus and stress. First, stress may be a predisposing risk factor of tinnitus^4^,^5^,^6^. In-patients with tinnitus indicated more stress than a normative healthy control group^1^ and reported more frequent and more stressful life events than a clinical control group^6^. Stress also seems to be an important factor in the transition from mild to severe tinnitus^7^. Second, cognitive maladjustment to stressful situations could favor tinnitus onset and maintenance^8^,^9^. In accordance, tinnitus patients used more maladaptive coping strategies than a clinical control group^6^. Neuroticism, a personality factor associated with increased experienced levels of anxiety, sadness, embarrassment, and guilt, has also been identified as a risk factor for tinnitus severity^10^. Lastly, tinnitus symptoms themselves may act as a stressor, resulting in higher general physiological arousal and psychological distress^11^. Consistent with this idea, research showed elevated ratings of subjective strain in response to experimentally induced stress in tinnitus patients compared to controls^12^.

About 10–15% of the general population is affected by tinnitus and the majority habituates to it^13^,^14^. Only in about 2–6% of all tinnitus patients the symptoms cause a considerable amount of psychological distress interfering with their lives^14^,^15^. The psychological understanding of why tinnitus becomes problematic in a small proportion of patients suggests that in those cases, it acquires a negative emotional significance through maladaptive cognitive appraisal and dysfunctional processes in the autonomic nervous system (ANS). Thus, the negative, catastrophic interpretations of the tinnitus percept are sustained and habituation to the tinnitus fails^8^,^9^,^16^. Central to cortical mechanisms associated with tinnitus annoyance is the limbic system. The amygdala, essential for the emotional evaluation of sensory stimuli and in turn also a control structure of the ANS, may be the essential common correlate in the maintenance of tinnitus annoyance and its bodily consequences, e.g. elevated levels of arousal^17^,^18^,^19^. This idea is supported by the finding that temporarily inactivating the amygdala can transiently reduce tinnitus annoyance^20^. Additionally, imaging studies demonstrated that limbic structures, including the amygdala, are frequently altered in tinnitus patients^21^,^22^,^23^,^24^.

Especially the sympathetic branch of the ANS plays an important role in stress and its chronic hyperactivity also seems to be involved in tinnitus^25^. This idea is corroborated by the old finding that sympathectomy can relieve tinnitus symptoms in patients with Ménière’s disease^26^. Recent studies have observed a positive association between tinnitus-related distress and sympathetic tone^27^ and a negative association between tinnitus-related distress and parasympathetic tone^28^. Also, successful suppression of tinnitus has been associated with an increase in parasympathetic tone^29^. Reports from tinnitus patients indicating higher levels of muscle tension in face, jaw and shoulder in contrast to healthy controls may provide a further hint for hyperarousal in tinnitus, even if this relationship may also be driven by direct somatosensory-auditory interactions^30^,^31^,^32^. Finally, the frequent co-occurrence of insomnia^33^,^34^ and depression^35^ with tinnitus might be mediated by hyperarousal^19^.

Taken together, autonomic functioning may be dysregulated in patients with chronic tinnitus, plausibly associated with a state of chronic stress^16^. In accordance, psychoendocrine research showed attenuated biological stress reactions measured by cortisol levels in tinnitus patients^36^,^37^,^38^. In contrast, one further study could find no altered psychophysiological stress reactivity in tinnitus as assessed with electromyography and skin conductance level^12^.

Recent psychophysiological research has advanced the role of heart rate variability (HRV) as another potent indicator of ANS activity and stress in healthy and clinical samples^39^,^40^,^41^. HRV describes the phenomenon that the duration of intervals between consecutive heartbeats (RR intervals) is subject to spontaneous oscillations^42^. Alterations in RR intervals may occur before any appreciable change occurs in HR itself^43^,^44^. Sympathetic activity induces an increase in HR with a concomitant decrease in HRV, while parasympathetic activity leads to a lower HR and a simultaneous increase in HRV. The two systems are constantly interacting to ensure adaptability^41^,^45^. Global HRV is supposed to reflect the interplay of influences from the sympathetic and parasympathetic branch^41^,^46^. Generally, high HRV is considered neurologically healthy^47^. Under acute stress, HR increases, HRV decreases transiently, and high correlations between the reduction in HRV and the perceived stress are observed in healthy samples^41^,^48^,^49^. Hence, HRV is considered an important marker of psychological stress^45^.

Few studies have used HR or HRV as markers of basal ANS functioning in chronic tinnitus. Two studies reported decreased HRV and a relative predominance of the sympathetic branch of the ANS in tinnitus patients^27^,^28^, whereas one further study did not detect differences in basal ANS activity between controls and elderly tinnitus patients^50^. We are not aware of any study that has assessed tinnitus patients’ ANS reactivity, i.e. stress response, with measures of HR and HRV. However, provocation tests are an essential aspect for stress assessment^51^. Moreover, increases of subjectively perceived stress are associated with increased tinnitus loudness^52^. Therefore, in the present study we initially used HR and HRV to investigate the ANS reactivity in patients with chronic tinnitus and healthy participants without tinnitus (controls). We examined ANS activity at rest and the ANS response to stress in two different stress tasks (arithmetic stress and concentration on the own tinnitus). Relaxation/resting phases after each stress task served to investigate the recovery from stress. We included subjective ratings of the perceived stressfulness of each experimental condition to compare physiological data with subjective strain. Additionally, tinnitus patients indicated the extent of the perceived presence of their tinnitus during each condition.

## Method and Materials

### Participants

Patients with chronic tinnitus out of the database of the Multidisciplinary Tinnitus Center Regensburg were contacted via postal mail and invited to participate in the study. Controls were recruited from the local community via adverts and selected by matching for age, sex, and education. Exclusion criteria were cardiovascular disease or diseases with influence on the autonomic nervous system (e.g. diabetes), intake of psychotropic medication (e.g. antidepressants) and other medication known to alter autonomic functioning up to four weeks before the study and regular usage of illegal drugs. Tinnitus was subjective and chronic in all participants with tinnitus, i.e. had been present for at least six months (mean duration = 139.42 months, *SD* = 113.44). Tinnitus was perceived monaurally in 15.8% and binaurally in 84.2% of patients with chronic tinnitus. Tinnitus was described as a tone (84.2%) or noise (15.8%). Tinnitus patients indicated poor hearing (21.1%), noise sensitivity (21.1%), both (31.6%) or no further ear-related problems (26.3%).

Participants gave written informed consent and the study was approved by the Ethics Committee of the University Hospital of Regensburg (reference number: 16–101–0037) and carried out in accordance with the approved guidelines.

Forty-one participants participated in the study. Each participant was tested in an individual session and was compensated with 20€. Two persons were excluded from the final analysis due to difficulties in carrying out the arithmetic stress task. Another participant was excluded because the electrocardiogram (ECG) showed ectopic beats. All three dropouts were patients with chronic tinnitus. The final sample comprised 38 persons, ages 21 to 70 (*M* = 49.32, *SD* = 11.49, 42% female), with 19 persons in each experimental group. Table 1 summarizes sample characteristics. No significant differences in age, sex, education, weight, caffeine consumption, smoking, and alcohol consumption were found. Thus, the comparability of the groups was given. To rule out the possibility that the descriptively higher cigarette consumption in the tinnitus group had influenced our data, we calculated all analyses excluding the smokers in the tinnitus group (*n* = 3) and the control group (*n* = 3), which did not change our results.

### Procedure

**Figure 1:**
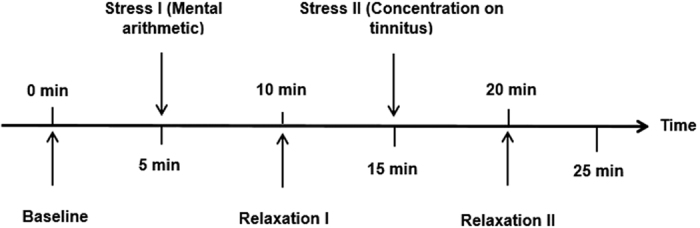
Experimental procedure with five experimental conditions (baseline, stress I, relaxation I, stress II, relaxation II). For each phase of 5 min, a separate ECG recording was gathered.

The experiment took place at the Department of Psychiatry and Psychotherapy of the medbo Regensburg from March 2016 till April 2016. Participants were asked not to drink caffeinated beverages or smoke up to one hour before the start of the experiment. Participants underwent a screening interview for personal data, medical history and consumption habits. Tinnitus patients were additionally interviewed about laterality of their tinnitus, type of tinnitus percept, and further problems with their ears. After that, participants were familiarized with the arithmetic stress task. Then, they filled out various questionnaires (Major Depression Inventory (MDI^53^; German version^54^), Perceived Stress Questionnaire 20 (PSQ-20^55^; German version^56^), NEO-Five-Factor-Inventory (NEO-FFI^57^; German version:^58^), Mehrfachwahl-Wortschatz-Intelligenztest for estimation of intelligence (MWT-B^59^), Tinnitus Questionnaire (TQ^8^; German version^60^). Next, participants were seated comfortably in a chair and sensors for HRV assessment were attached. They were instructed not to speak and to restrict all voluntary movements to a minimum during the ECG recordings. The experimental procedure is illustrated in Fig. 1. First, participants underwent a resting measurement with a duration of five minutes (baseline). They were asked to relax as much as possible without closing their eyes to ensure comparability across conditions. Afterwards, the first stress measurement (stress I) of five minutes took place during which participants conducted the arithmetic stress task. Subjects were asked not to communicate their reaction to the stress tasks overtly (e.g. cursing) to prevent interference with ECG data collection. After that, a relaxation phase (relaxation I) identical to the baseline measurement took place. For the second stress measurement (stress II), participants were given earplugs to shield themselves from any noise present in the room. Tinnitus patients were instructed to concentrate on their tinnitus percept, while controls were asked to listen to noises possibly observable in their ears. After five minutes, earplugs were removed and participants passed a final relaxation phase (relaxation II) of five minutes. For each experimental condition, a separate ECG recording was gathered. After each condition, participants had to indicate their subjective level of perceived stress (“How stressed have you been feeling during the last five minutes?”) verbally on a 11-point Likert scale (0 = not at all; 10 = very much). Participants with chronic tinnitus also indicated the subjective perception of the tinnitus during each of the five measurements (“How many percent of the time have you been consciously aware of your tinnitus in the last five minutes?”). After the ratings for the second relaxation phase were obtained, sensors for data recording were removed. The entire experiment took about 75–90 minutes.

### Arithmetic Stress Task

Mental arithmetic, a mild, but effective environmental stressor previously used in healthy^41^,^61^ and clinic samples^12^,^62^ was administered to induce stress in participants. To minimize the effects of speaking on HRV^61^, the arithmetic stress task was carried out silently on a computer screen (HP, Type 1702, screen width 17”) that was positioned approximately one meter in front of the participants. The task was written with the stimulus-delivery software Presentation, version 0.71 (Neurobehavioral Systems, Berkeley, CA, USA). Information was presented in white font (font size: 29 pt) on a black screen. Participants were presented with arithmetic problems that required adding or subtracting the number 17 to or from two- or three-digit figures (e.g. 176 + 17; 61–17) which was determined as a potent stressor in preliminary tests in our laboratory. After the arithmetic problem had appeared in the center of the screen for 1000 ms, the screen turned black for 4000 ms and participants had to solve the problem. Using a wireless keyboard on their laps, participants had to select the correct solution from two alternatives that were presented simultaneously for 1000 ms by pressing either the left or right arrow key corresponding to the locations of the solutions on the screen. Immediately after their response or a maximum of 1000 ms, trial-to-trial feedback of 1000 ms was provided as to if the participant had chosen the right solution (“Correct”), the wrong solution (“Incorrect”) or took too long (>1000 ms) to answer (“Too slow”). The next trial started automatically after feedback was delivered. The arithmetic stress task with a total of 45 arithmetic problems lasted for 5 min. A training session with five simple items and verbal instructions delivered by the experimenter at the beginning of the experiment (before electrode installation) ensured that participants understood the task and the stimulus response-assignments.

### ECG recordings and data analysis

ECG data were recorded using four silver/silver chloride (Ag/AgCl) surface ring electrodes (EASYCAP GmbH, Herrsching, Germany) with an outer diameter of 1.2 cm and an inner diameter of 0.6 cm. The ECG signal was amplified and digitized with a BrainAmp DC amplifier linked to BrainVision Recorder software (both Brain Products GmbH, Gilching, Germany). The sampling rate was set at 1000 Hz. After cleansing the participant’s skin with isopropyl alcohol (70%), two active electrodes were attached beneath the left and right clavicle. A reference electrode and a ground electrode were attached in the participant’s neck area. The electrodes were covered with electrode cream (Grass, Warwick, RI, USA) to ensure good conductivity.

For preliminary off-line data processing, BrainVision Analyzer (Brain Products GmbH, Gilching, Germany) was used. A high pass filter of 15 Hz and a low pass filter of 45 Hz were implemented, as well as a Notch filter (50 Hz). The recorded data were re-referenced to an averaged ECG signal using linear derivation (left minus right clavicle electrode). For subsequent HRV data analysis according to the guidelines recommended by the Task Force of the Society of Cardiology (1996), we used Kubios HRV^63^. All recordings were visually examined and manually corrected for artifacts. In two cases, artifacts caused by coughing were located at the beginning or ending of the recording, so only the artifact-free period was analyzed. For one recording with high rates of voluntary movement, different filter settings (high pass: 15 Hz, low pass: 25 Hz) were applied in preliminary data processing to be able to obtain RR intervals series from the data.

Two essential measures in the time domain are mean HR, which is indexed in beats per minute, and the standard deviation of normal to normal RR intervals (SDNN), which is given in units of ms. SDNN estimates global HRV^44^,^64^,^65^. We calculated mean HR and SDNN for each subject in each of the five experimental conditions. Further analyses using frequency domain measures of HRV (low- and high-frequency power) showed results identical to SDNN and are not reported in this paper.

### Design

A two (group factor: tinnitus group vs. control group) by five (condition: baseline, stress I, relaxation I, stress II, and relaxation II) mixed factorial design with repeated measures on the experimental condition variable was administered. Dependent variables were mean HR and global HRV (SDNN) as well as subjective ratings of stress. Additionally, a one-way repeated-measures ANOVA was conducted to explore the effect of condition on the perceived presence of tinnitus during measurements.

### Statistical analysis

Statistical analyses were performed with SPSS 22.0 (SPSS/IBM, Chicago, IL, USA). The significance threshold was set at *p* = 0.05 for the statistical tests. Greenhouse-Geisser-correction was applied if the assumption of sphericity was violated in repeated-measures analysis of variance (ANOVA). Bonferroni correction was used for pairwise comparisons. Effect sizes for the *t*-tests are reported with Cohen’s *d* calculated with G*Power software, version 3.1^66^. Exploratory correlational analyses for physiological and psychological measures of stress were conducted using Pearson’s *r*. Scatterplots served to detect outliers.

## Results

### Arithmetic stress task

On average, the tinnitus group solved 25.74 (*SD* = 5.01) of the 45 arithmetic problems presented on the arithmetic stress task correctly. The mean number of correct responses in the control group was similar (*M* = 27.63, *SD* = 5.86). The performances did not differ significantly (*t* < 2).

### ECG data

#### Mean HR

**Figure 2:**
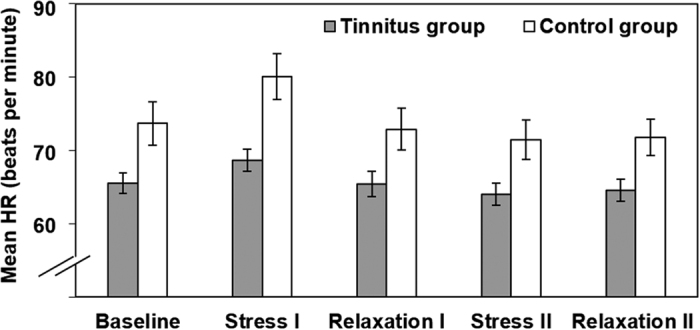
Mean HR on the five experimental conditions (baseline, stress I, relaxation I, stress II, relaxation II) as a function of group status. Error bars represent standard errors of the means.

Results for mean HR are presented in Fig. 2. Mean HR data was submitted to a mixed-design 2 (group factor) × 5 (condition) ANOVA. Mauchly’s test indicated that the assumption of sphericity had been violated, χ^2^(9) = 56.08, *p* < 0.001, therefore degrees of freedom were corrected using Greenhouse-Geisser estimates of sphericity (ε = 0.52). The ANOVA revealed a main effect of group factor, *F*(1, 36) = 7.07, *p* = 0.012, η_p_^2^ = 0.16, which indicated that, overall, mean HR was lower for patients with chronic tinnitus than for controls. The analysis also yielded a main effect of condition, *F*(2.07, 74.46) = 39.65, *p* < 0.001, η_p_^2^ = 0.52. The interaction between group factor and condition was also significant, *F*(2.07, 74.46) = 4.31, *p* = 0.016, η_p_^2^ = 0.11. Bonferroni-corrected post hoc analyses confirmed that controls displayed a significantly higher mean HR during stress I (*p* = 0.015, *d* = 1.06). None of the other comparisons reached significance level (all *p*s > 0.10.) Thus, the interaction effect can be explained by mean HR values during stress I where tinnitus patients showed specifically lower mean HR than did controls. Overall in both groups, during stress I, i.e. the arithmetic stress task, mean HR was significantly higher than during all other conditions phases (all *p*s < 0.001). Hence, stress induction using mental arithmetic was successful. Additionally, stress II, i.e. attention allocation to tinnitus/ear noises, differed significantly from baseline (*p* = 0.002) and relaxation I (*p* = 0.006). None of the other comparisons reached significance level (all *p*s > 0.114).

#### SDNN

A 2 (group factor) × 5 (condition) ANOVA was conducted on SDNN data. Neither the group factor nor the experimental condition had a statistically significant impact on SDNN data (*F*s < 2). The interaction of group factor × condition did not reach significance level, either (*F* < 1).

#### Correlation of mean HR and SDNN

We correlated mean HR with SDNN data for the two groups separately. In the control group, there was a trend towards statistical significance for the association of HR and SDNN during baseline, *r*(19) = −0.26, *p* = 0.143, relaxation I, *r*(19) = −0.21, *p* = 0.198, stress II, *r*(19) = −0.23, *p* = 0.169, and relaxation II, *r*(19) = −0.30, *p* = 0.107. In the tinnitus group, the association of HR and SDNN trended towards statistical significance during relaxation I, *r*(19) = 0.31, *p* = 0.101. No other trends or statistically significant relationships were found (all *p*s > 0.389).

### Verbal Ratings

#### Subjective stress ratings

**Figure 3:**
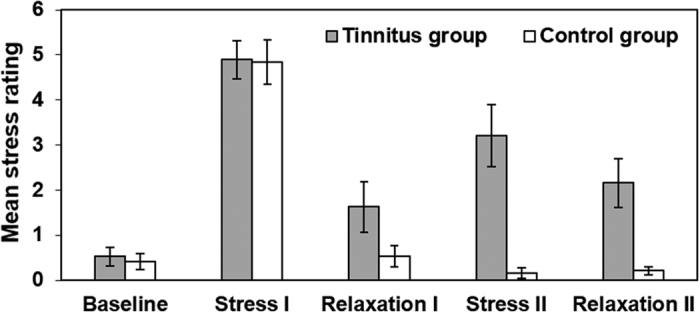
Mean stress ratings on the five experimental conditions (baseline, stress I, relaxation I, stress II, relaxation II) as a function of group status. Error bars represent standard errors of the means.

Figure 3 illustrates subjective stress ratings after each experimental condition for tinnitus patients and controls. These results were submitted to a 2 (group factor) × 5 (condition) ANOVA. Mauchly’s test indicated that the assumption of sphericity had been violated, χ^2^(9) = 30.00, *p* < 0.001, therefore degrees of freedom were corrected using Greenhouse-Geisser estimates of sphericity (ε = 0.74). The analysis revealed a significant main effect of group, *F*(1, 36) = 10.37, *p* = 0.003, η_p_^2^ = 0.22, which indicated that, overall, tinnitus patients perceived all five conditions as more stressful than did controls. Additionally, there was a significant main effect of condition, *F*(2.94, 105.9) = 53.68, *p* < 0.001, η_p_^2^ = 0.60. However, these main effects were qualified by a significant group × condition interaction, *F*(2.94, 105.9) = 7.22, *p* < 0.001, η_p_^2^ = 0.17.

On average, a trend towards higher subjective stress was reported by tinnitus patients than by controls during relaxation I, but the comparison did not reach significance, *t*(36) = 1.83, *p* = 0.076, *d* = 0.60. Subjective stress ratings for stress II were significantly higher in the tinnitus group than in the control group, *t*(18.56) = 4.39, *p* < 0.001, *d* = 1.42. Additionally, relaxation II was perceived as significantly more stressful by tinnitus patients than by controls, *t*(19.81) = 3.47, *p* = .002, *d* = 1.13. None of the other comparisons reached significance level (all *t*s < 1). Bonferroni-corrected pairwise comparisons confirmed that in both groups, stress ratings for stress I were significantly higher than for all other four conditions (all *p*s < 0.001), providing further evidence that stress induction using mental arithmetic was successful. In addition, ratings after baseline differed significantly from ratings after stress II (*p* = 0.011). None of the other comparisons reached significance (*p*s > 0.236).

#### Perceived presence of tinnitus

**Figure 4:**
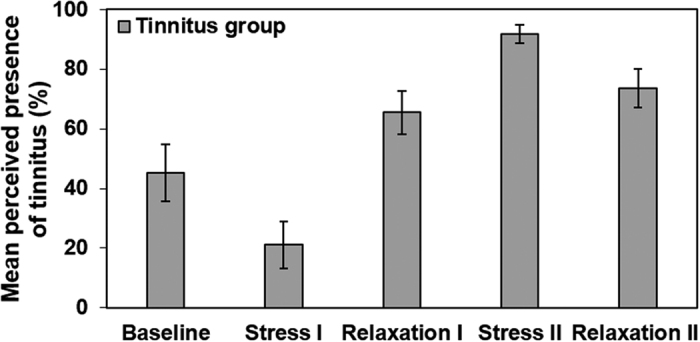
Mean perceived temporal tinnitus presence on the five conditions (baseline, stress I, relaxation I, stress II, relaxation II). Error bars represent standard errors of the means.

Results for the ratings of the perceived temporal presence of tinnitus during each condition are shown in Fig. 4. A one-way repeated measures ANOVA was conducted to examine the effect of condition on the perceived presence of tinnitus. The assumption of sphericity was violated as indicated by Mauchly’s test, χ^2^(9) = 17.82, *p* = 0.045, thus degrees of freedom were corrected using Greenhouse-Geisser estimates of sphericity (ε = 0.69). The ANOVA showed that ratings of the perceived presence of tinnitus changed within the conditions, *F*(2.76, 49.68) = 26.63, *p* < 0.001, η_p_^2^ = 0.59.

Bonferroni-corrected pairwise comparisons indicated that the perceived presence of the tinnitus during stress I was in both groups significantly lower than in relaxation I (*p* = 0.001), stress II (*p* < 0.001) and relaxation II (*p* < 0.001). Additionally, tinnitus was perceived more extendedly in stress II than during baseline (*p* < 0.001), relaxation I (*p* = 0.002) and relaxation II (*p* = 0.006). Moreover, the perceived presence of tinnitus was higher during relaxation II than during baseline (*p* = 0.016). None of the other comparisons reached significance level (*p*s > 0.060).

### Correlation analyses

#### Stress ratings and HRV data

To explore the association between subjective acute strain and physiological stress reactivity, we correlated self-reported stress ratings with the respective ECG data (mean HR, SDNN) for the two groups separately. There were no significant correlations between stress ratings and mean HR. HRV measured with SDNN was negatively correlated with strain ratings during the mental arithmetic task in the control group, indicating that a reduction in HRV was associated with higher subjective strain, *r*(19) = −0.47, *p* = 0.042. In the tinnitus group, no such association was found neither for the arithmetic stress task nor the stressor “attention allocation to the own tinnitus”.

#### TQ score and HRV data

For the tinnitus group, correlation analyses were used to explore relationships between tinnitus severity as measured with TQ scores and HRV data (mean HR, SDNN). Only non-significant relationships were found.

#### TQ and subjective stress ratings

Correlations between TQ scores and subjective stress ratings were analyzed. TQ scores were positively correlated with stress ratings for the first relaxation phase, *r*(19) = 0.62, *p* = 0.005. None of the other correlations reached significance level.

## Discussion

In this study, we investigated the stress reactivity in patients with chronic tinnitus using HR and HRV. Contrary to previous studies^27^,^28^, tinnitus patients and control subjects did not differ significantly in baseline HRV. These results challenge the assumption of a greater basal arousal in patients with chronic tinnitus^19^. Unexpectedly, baseline levels of mean HR were significantly lower in tinnitus patients than in controls. We expected increased baseline HR levels in tinnitus sufferers as it is known from patients with PTSD^67^, another disease with hyperarousal as major symptom. It is possible that hyperarousal affects tinnitus patients not as much as PTSD patients. Additionally, as exclusion criteria in the present study were stringent to control for confounders of HRV, the tinnitus sample was rather homogeneous and tinnitus severity was lower than in other investigated clinical tinnitus samples (e.g.^68^). The rather low tinnitus severity in our sample may have been the reason why we did not detect HRV alterations. Yet, our data are in line with a recent study that reported no group differences in HRV, but significantly lower mean HR in the tinnitus group, using 24h-Holter-recordings to explore ANS functioning in an elderly tinnitus population^50^. Decreased HR in patients with chronic tinnitus may be interpreted as physiological long-term adaptation of the cardiac system to mild chronic stress, comparable to competitive sports where trained athletes show decreased HR compared to untrained controls^69^. Psychoendocrine research has suggested tinnitus as a chronic stressor resulting in diminished efficiency of the stress hormone system^36^,^38^.

Relative to healthy controls, tinnitus patients displayed a lower mean HR increase during the first stress task. This was confirmed by the significant interaction effect of group factor × condition for mean HR and post hoc analyses that illustrated that group differences were greatest during stress I. Mean HR therefore suggests that tinnitus patients’ response to stress was attenuated, indicating maladaptive ANS reactivity. Similarly, previous studies showed blunted cortisol stress responses in patients with chronic tinnitus^36^,^37^,^38^. Contrary to HR, HRV did not reflect stress exposure – neither in the patient group nor in the control group – even though HRV decreased in both groups on a descriptive, statistically not significant level in response to the arithmetic stress task.

These findings suggest that HRV may not be a valid marker of stress. The reliability of short-term measures of HRV has been questioned^70^. Moreover, HRV has been shown to be prone to confounding variables such as gender, age, weight, smoking, depression, medication of antidepressants, antihypertensive drugs and antiarrhythmic drugs, and importantly, HR itself^42^,^71^,^72^. Successful matching and preliminary screening ensured that these confounding variables were controlled for in our study sample, except for HR, which differed significantly between the groups. It is possible that group differences in HR prevented us from detecting subtler differences in HRV. Higher HR has been associated with lower HRV^47^. We observed this trend in the control group during four phases of measurement. In the tinnitus group, no associations were found, except for relaxation I, where the trend was opposed to common observations, i.e. a higher HR was associated with higher HRV. Speculatively, the reduced HR and the reversal or lack of correlation between HR and HRV reflect a dysfunction of the ANS in tinnitus patients resulting in abnormal regulation of physiological variables observed in healthy controls, i.e. increase in HR with concomitant decrease in HRV in response to stress.

Subjective perceived stress was increased during the relaxation phases and stress II in tinnitus patients compared to controls, whereas the arithmetic stress tasks induced similar levels of subjective stress in both groups. Tinnitus presence may be a factor influencing the subjective stress experience in tinnitus patients. Ratings for the perceived tinnitus presence indicated that during arithmetic stress exposure, patients were distracted from their tinnitus percept and stress levels were similar to the ones of controls. During the following relaxation phase, the tinnitus was subjectively perceived for a longer amount of time due to a discontinuation of distraction and simultaneous re-focusing on the tinnitus percept and stress levels remained high. When tinnitus patients should focus on their tinnitus percept during the second stress phase, no distraction was given and ratings for the perceived tinnitus presence further increased in comparison to arithmetic stress exposure. In the subsequent relaxation phase, tinnitus presence and stress levels again stayed high. The lack of decline in stress ratings during the relaxation phases may signal disturbed recovery from stress in tinnitus patients. Increased tinnitus presence after the stress tasks is in line with anecdotal reports of many tinnitus patients who indicate that tinnitus presence is augmented after phases of intensified stress or physical activity. One could speculate that subjective strain after stress may take longer to abate because physiological adaptation to stress fails during stress exposure. Also, stress ratings for the first relaxation phase and long-term tinnitus severity were positively correlated, indicating that increased levels of life strain related to tinnitus went along with higher levels of experienced stress during relaxation. Negative cognitive appraisal mechanisms associated with chronic tinnitus, e.g. intensified attention allocation to the tinnitus, may impair recovering from acute stress.

Contrary to healthy controls in our and other samples^41^,^48^,^49^, a lack of correlation between subjective strain ratings and objective physiological data emerged in tinnitus patients, as has been shown previously^12^. Similarly, there were no significant correlations between chronic tinnitus severity as indexed by TQ scores and physiological data, which also parallels earlier findings^12^. These findings suggest that physiological stress reactions and the subjective reported short-term and long-term stress perception are de-synchronized, again indicating disturbed physiologic reactivity in chronic tinnitus. Speculatively, some sort of cognitive bias predisposes tinnitus patients to think more negatively about relatively small changes in arousal, enhancing subjective strain, yet the exact mechanisms are still unclear.

Taken together, our results of reduced baseline HR, reduced HR increase during the arithmetic stress task, decoupling of HR and HRV and de-synchronized physiological and subjective perceived stress reactivity confirm a dysfunction of the ANS in chronic tinnitus, as postulated in psychological^8^,^9^ and neurophysiological^17^,^24^,^73^ models of chronic tinnitus. Unexpectedly, we were not able to identify hints for ANS hyperarousal in tinnitus patients questioning the validity of HRV as marker for stress reactivity in patients with mild to moderate chronic tinnitus. All our findings suggest a rather blunted ANS activity in the investigated sample. Furthermore, we found subjectively increased stress and tinnitus presence levels after stress exposure, which reflects very well the clinical complaints of many tinnitus patients. Further research is needed to elucidate whether the blunted physiological activity is related to the observed impaired relaxation after stress exposure.

Our findings also demonstrated that physiological findings concerning ANS functioning in chronic tinnitus seem to be more subtle and less uniform than assumed so far, providing a further hint that tinnitus is a pathophysiological heterogeneous condition. More research involving large samples will be needed to better understand the interplay between tinnitus severity, subjective stress and ANS function.

## Additional Information

**How to cite this article**: Betz, L. T. *et al*. Stress reactivity in chronic tinnitus. *Sci. Rep.* **7**, 41521; doi: 10.1038/srep41521 (2017).

**Publisher's note:** Springer Nature remains neutral with regard to jurisdictional claims in published maps and institutional affiliations.

## Figures and Tables

**Table 1:** Clinical and Demographic Characteristics.

Measure	Tinnitus patients (*n* = 19)	Controls (*n* = 19)	Statistics
Age in years (*SD*)	47.89 (12.61)	50.74 (10.39)	*t* < 1
Sex	37% female	47% female	χ^2^ < 1
Education level	47% A-level	53% A-level	χ^2^ < 1
Weight in kg (*SD*)	79.53 (15.54)	79.42 (12.85)	*t* < 1
Units of caffeinated beverages consumed per week (*SD*)	16.37 (10.18)	18.58 (13.23)	*t* < 1
Cigarettes consumed per week (*SD*) (*n* = 3 smokers per group)	19.53 (46.64)	4.18 (12.16)	*t* < 2
Units of alcohol consumed per week (*SD*)	2.38 (3.55)	3.40 (3.58)	*t* < 1
MDI score (*SD*)	7.74 (6.58)	3.79 (2.74)	*t*(24.06) = 2.41, *p* = 0.024, *d* = 0.78
PSQ-20 score (*SD*)	39.56 (19.48)	20.09 (9.93)	*t*(26.77) = 3.88, *p* = 0.001, *d* = 1.26
NEO-FFI: N score (*SD*)	1.71 (0.72)	0.94 (0.60)	*t*(36) = 3.56, *p* = 0.001, *d* = 1.16
NEO-FFI: E score (*SD*)	2.18 (0.51)	2.46 (0.51)	*t* < 2
NEO-FFI: O score (*SD*)	2.39 (0.49)	2.34 (0.61)	*t* < 1
NEO-FFI: A score (*SD*)	2.57 (0.41)	2.82 (0.59)	*t* < 2
NEO-FFI: C score (*SD*)	3.00 (0.53)	2.92 (0.58)	*t* < 1
MWT-B score (*SD*)*	28.68 (3.32)	32.42 (2.80)	*t*(36) = 3.76, *p* = 0.001, *d* = 1.22
TQ score (*SD*)		22.11 (12.44)	

^*^MWT-B score added to the analyses as a covariate did not change our results.
